# A Defective Crosstalk Between Neurons and Müller Glial Cells in the *rd1* Retina Impairs the Regenerative Potential of Glial Stem Cells

**DOI:** 10.3389/fncel.2019.00334

**Published:** 2019-07-25

**Authors:** Yanel A. Volonté, Harmonie Vallese-Maurizi, Marcos J. Dibo, Victoria B. Ayala-Peña, Andrés Garelli, Samanta R. Zanetti, Axel Turpaud, Cheryl Mae Craft, Nora P. Rotstein, Luis E. Politi, Olga L. German

**Affiliations:** ^1^Instituto de Investigaciones Bioquímicas de Bahía Blanca, Departamento de Biología, Bioquímica y Farmacia, Universidad Nacional del Sur – National Research Council of Argentina (CONICET), Bahía Blanca, Argentina; ^2^Department of Ophthalmology, USC Roski Eye Institute, Keck School of Medicine of the University of Southern California, Los Angeles, CA, United States; ^3^Department of Integrative Anatomical Sciences, Keck School of Medicine of the University of Southern California, Los Angeles, CA, United States

**Keywords:** Müller glial cells, stem cells, retinal degeneration, retinal regeneration, photoreceptors

## Abstract

Müller glial cells (MGC) are stem cells in the retina. Although their regenerative capacity is very low in mammals, the use of MGC as stem cells to regenerate photoreceptors (PHRs) during retina degenerations, such as in retinitis pigmentosa, is being intensely studied. Changes affecting PHRs in diseased retinas have been thoroughly investigated; however, whether MGC are also affected is still unclear. We here investigated whether MGC in *retinal degeneration 1* (*rd1*) mouse, an animal model of retinitis pigmentosa, have impaired stem cell properties or structure. *rd1* MGC showed an altered morphology, both in culture and in the whole retina. Using mixed neuron-glial cultures obtained from newborn mice retinas, we determined that proliferation was significantly lower in *rd1* than in *wild type (wt)* MGC. Levels of stem cell markers, such as *Nestin* and *Sox2*, were also markedly reduced in *rd1* MGC compared to *wt* MGC in neuron-glial cultures and in retina cryosections, even before the onset of PHR degeneration. We then investigated whether neuron-glial crosstalk was involved in these changes. Noteworthy, *Nestin* expression was restored in *rd1* MGC in co-culture with *wt* neurons. Conversely, *Nestin* expression decreased in *wt* MGC in co-culture with *rd1* neurons, as occurred in *rd1* MGC in *rd1* neuron-glial mixed cultures. These results imply that MGC proliferation and stem cell markers are reduced in *rd1* retinas and might be restored by their interaction with “healthy” PHRs, suggesting that alterations in *rd1* PHRs lead to a disruption in neuron-glial crosstalk affecting the regenerative potential of MGC.

## Introduction

Retinitis pigmentosa constitutes a group of inherited diseases that have as a common feature the degeneration and loss of rod and cone photoreceptors (PHRs). Although the genetic mutations leading to this disease are well-established, it has still no cure or effective treatments. The use of stem cells to replace neuronal loss in the injured retina is now being actively investigated as a promising new treatment for this disease. The discovery that Müller glial cells (MGC) can regenerate retina neurons following diverse insults (Karl et al., 2008) makes these cells potentially useful candidates for replacement therapies. MGC have multiple key roles in the retina, such as providing nutrients and trophic factors required for PHR survival (Bringmann et al., 2009; Vecino et al., 2016). Fischer and Reh (Fischer and Reh, 2003) have shown that MGC are stem cells that effectively regenerate the eye in fish and lower vertebrates. However, their regenerative potential is extremely low in vertebrates; generation of a few novel neurons in the murine retina was only possible because of the use of multiple exogenous stimuli (Fischer and Reh, 2003). *Notch* and *Wnt* signaling have been proposed to regulate the mechanisms by which MGC promote retina regeneration (Das et al., 2006). We previously showed that MGC express several stem cell markers and can transform PHR progenitor cells into multipotent stem cells, which in turn differentiate as functional PHRs (Insua et al., 2008; Simón et al., 2012). Nevertheless, MGC are inefficient in regenerating the retina in humans, such as in patients suffering from retinitis pigmentosa, and in the *rd1* mice, a frequently used animal model for this disease.

About 100 gene mutations have been identified to cause PHR cell death in retinitis pigmentosa (Hartong et al., 2006; Huang et al., 2017), most of them affecting PHRs. In the *rd1* mice, the occurrence of a mutation in the beta-subunit of rod cGMP phosphodiesterase gene, which mainly affects PHRs, is responsible for the disease (Pittler and Baehr, 1991). By contrast, only a few mutations causing the disease have been found in MGC; one of them, affecting a gene encoding CRALBP in an autosomal recessive form of retinitis pigmentosa, may disturb Vitamin A metabolism (Maw et al., 1997). Little is known regarding the role of MGC in this disease. Following neuronal damage in the injured retina, MGC show a reactive gliosis, characterized by an increase in glial cell proliferation, preceded by downregulation of the cell cycle blocker, p27 ^kip1^ (Dyer and Cepko, 2000; Bringmann et al., 2006). Defects in MGC have been reported in another ocular disorder, Retinal Telangiectasia; a mutation in *Crb1* gene delocalizes the cellular connections at retinal MGC/PHR junctions (Zhao et al., 2015). Even when these alterations in MGC may not be the main cause of retinal degenerations, they might disrupt the normal crosstalk between MGC and PHRs, thus contributing to the degenerative process (Dong et al., 2017).

Retinal degeneration in the *rd1* mice occurs much faster than in humans. In the healthy mouse retina, most PHRs are “born” between PN days (PND) 0 and 2; soon after, their progenitors exit the cell cycle and begin their differentiation; a small peak in their apoptosis is detected by PND 7 and 8, and no cell death is detectable thereafter (Young, 1984). In the *rd1* retina, significant PHR cell death starts by PND 11, peaking by PND 15 and 16 (Lolley et al., 1994; Portera-Cailliau et al., 1994); after 4 weeks, while in the healthy mice PHRs become differentiated and functional, in the *rd1* mice the PHR cell layer completely disappears (Farber and Lolley, 1974; Politi and Adler, 1988).

The finding of the group of Canto Soler that human induced pluripotent stem cells (iPS) differentiate into retinal progenitors that originate retinal tissue containing the major retinal cell types, including functional PHRs (Zhong et al., 2014), underscores the therapeutic potential of stem cells. However, whether MGC are adequate sources of stem cells for retina regeneration in mammals remains to be established. In particular, alterations in PHRs in retinitis pigmentosa might change their normal interactions with MGC, disturbing neuron-glial crosstalk; in turn, this would lead to a deficient release of survival factors by MGC and contribute to a generalized retinal damage. Hence, understanding the significance of the alterations in MGC and in neuron-glial crosstalk during retina degeneration is particularly relevant when considering the use of MGC as a source of stem cells in regenerative therapies.

To establish whether MGC isolated from diseased retinas are a viable option for replacement therapies it is necessary to ascertain if these cells differ in their regenerative potential and in their cellular interactions with PHRs in healthy and degenerating retinas. For this purpose, we first analyzed stem cell characteristics of MGC in whole retinas and neuron-glial cultures from *rd1* and *wt* animals. Then, we separately prepared pure neuronal and MGC cultures to obtain co-cultures and investigated the interactions of *rd1* MGC with either *rd1* or *wt* neurons. We determined that proliferation and the levels of stem cell markers decreased in *rd1* MGC compared to their *wt* counterparts, and that these markers were partially restored when *rd1* MGC were co-cultured with *wt* neurons, suggesting that an impaired neuron-glial crosstalk affects the stem cell potential of *rd1* MGC.

## Materials and Methods

### Materials

Two-day-old C57BL/6J (*wt)* or C57BL/6J-Pde6brd1-2J/J (*rd1)* mice bred in our own colony were used in all experiments. All proceedings concerning animal use were in accordance with the guidelines published in the NIH *Guide for the care and use of laboratory animals* and following the protocols approved by the Institutional Committee for the Care of Laboratory Animals from the Universidad Nacional del Sur (Argentina). Plastic 35-mm-diameter and 60-mm-diameter culture dishes were purchased from Greiner Bio-One (GBO). Fetal bovine serum (FBS) was from Natocor (Córdoba, Argentina). Dulbecco’s modified Eagle’s medium (DMEM) was purchased from Life Technologies (Grand Island, NY, United States). Trypsin, trypsin inhibitor, transferrin, hydrocortisone, putrescine, insulin, polyornithine, selenium, gentamycin, 4,6-diamidino-2-phenylindole (DAPI), poly-ornithine, paraformaldehyde (PF) and primers for RQ-PCR were from Sigma (St. Louis, MO, United States). Fluorescein-conjugated secondary antibodies were from Jackson InmunoResearch (West Grove, PA, United States). Type 2 collagenase was purchased from Invitrogen. Monoclonal antibodies against BrdU (clone G3G4) and VIMENTIN (clone 40E-C) were from DSHB (developed under the auspices of the NICHD and maintained by the University of Iowa, Department of Biological Sciences), NESTIN was from DSHB and from ABCAM (clone Rat-401), anti-Glutamine Synthetase (GS) and anti-SOX2 (SRY, sex determing region Y-box 2) were from ABCAM. Polyclonal goat anti-SOX2 (Santa Cruz # sc-17320) was a generous gift from Valeria Levi and Alejandra Guberman (University of Buenos Aires, Argentina). Polyclonal rabbit anti-CRX was from Cheryl M. Craft’s laboratory (Zhu and Craft, 2000). CellTracer^®^ Green CMFDA Dye, Alexa Fluor^TM^ 555 Phalloidin, TO-PRO^TM^-3 Iodide, terminal deoxynucleotidyl transferase (Tdt), BrdUTP (5-Bromo-2′-Deoxyuridine 5′-Triphosphate) and TdT Buffer were from Molecular Probes, Invitrogen (Carlsbad, CA, United States). Quick-zol RNA Isolation Reagent was from Kalium technologies (Embiotec). The Moloney Murine Leukemia Virus Reverse Transcriptase (M-MLV RT) and RNasin^®^ Ribonuclease Inhibitors were from Promega. KAPPA SYBRs FAST qPCR Kit were from Biosystems S.A. (Buenos Aires, Argentina). The IntraStain, Two-step fixation and permeabilization for Flow Cytometry was from Agilent Dako. OCT Embedding Compound Cryoplast^®^ was from Biopack^®^. Solvents were HPLC grade, and all other reagents were analytical grade.

### Retina Cultures

#### Müller Glial Cell Cultures

**FIGURE 1:**
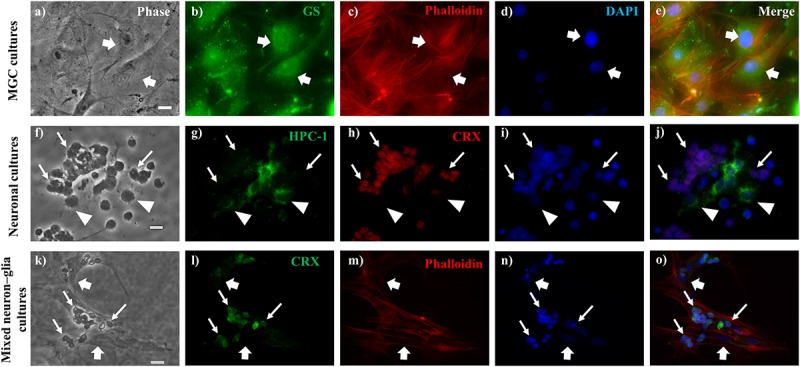
Identification of neurons and Müller glial cells (MGC) in glial, neuronal and mixed neuron-glial cultures. Phase **(a,f,k)** and fluorescence **(b–e,g–j,l–o)** photomicrographs of *wt* MGC culture **(a–e)**, 2-day *wt* neuronal cultures **(f–j)** and 6-day *wt* mixed neuron-glial cultures **(k–o)**, showing MGC (thick arrows) identified by their anti-Glutamine Synthase (GS) labeling **(b)**, with their actin cytoskeleton visualized by phalloidin labeling **(c,m)**; photoreceptors (thin arrows) labeled with anti-CRX **(h,l)** and amacrine neurons (arrowheads) labeled with HPC-1 **(g)** antibodies. Cell nuclei were visualized with the DNA probe DAPI **(d,i,n)**. Note that cell nuclei were markedly smaller in neurons than in MGC **(n)**. Merge images **(e,j,o)**. Scale bars: 20 μm.

Purified cultures of Müller glial cells were prepared following protocols previously described (Hicks and Courtois, 1990). Briefly, newborn mouse eyes were excised and incubated overnight in DMEM at room temperature and then treated with trypsin (1 mg/ml) and type 2 collagenase (2 mg/ml). Retinas were then dissected, chopped into small pieces, and seeded onto 35-mm-diameter plastic dishes, in culture medium supplemented with 10% FBS. The medium was routinely replaced every 3–4 days. After 8–10 days, pure Müller glial cells became confluent and were used for neuron-glial co-cultures (Figures 1a–e).

#### Neuronal Cultures

Neuronal cultures from 2-day mouse retinas were obtained following previously described procedures, with slight modifications (Politi et al., 1996; Rotstein et al., 1996, 1997). In brief, after dissection and dissociation of the retinas, the cells were resuspended in a chemically defined medium (Politi et al., 1988) and seeded onto 35-mm-diameter plastic dishes, pretreated with poly-ornithine and schwannoma-conditioned medium (Adler, 1982). Cultures were incubated at 36°C in a humidified atmosphere of 5% CO_2_. Immediately after seeding, nearly all cells were undifferentiated retinal progenitor cells, which were still in the cell cycle; many of them underwent their last mitotic division during the first day *in vitro*. Soon after exiting the cell cycle, cells began to differentiate into PHRs and amacrine neurons (Figures 1f–j).

#### Mixed Neuron–Glial Cultures

To analyze the interactions between MGC and neurons from either *wt* or *rd1* retinas, we obtained long-term mixed cultures of MGC and retina neurons, following previously described protocols (Politi et al., 1996; Simón et al., 2012), with slight modifications. Briefly, retinas from 2-day-old mice were chemically and mechanically dissociated with trypsin [250 μl of 0.25% trypsin in 6 ml of calcium- and magnesium-free Hanks (CMF)] for 6 min. Cells were then resuspended in DMEM medium supplemented with 10% FBS and seeded onto 35-mm-diameter plastic dishes with no previous treatment. Analysis of MGC began at day 6, since only after this time *in vitro*, MGC are clearly recognizable and begin to express most of their markers. This time *in vitro* is equivalent to 8 days *in vivo*, when the retina has a maximum of gliogenic factors and cues that signal and stabilize glial fate (Nelson et al., 2011; Figures 1k–o).

#### Neuron-Glial Co-cultures

To investigate whether the crosstalk between neurons and glial cells might modulate the stem cell potential of MGC, we prepared neuron-glial co-cultures combining *rd1* and *wt* neurons with either *rd1* or *wt* MGC. Pure neuronal cultures from *wt* or *rd1* retinas were initially labeled with the fluorescent dye CellTracer^®^ following the manufacturer’s specifications and after 3 days *in vitro* they were co-cultured with unlabeled *rd1* or *wt* MGC for 24 h. For this purpose, confluent (8−10 days) *rd1* or *wt* MGC were detached from their substrata and reseeded over the pre-labeled pure neuronal cultures. As the dye fluorescence persists during the whole culture time, even after mitosis, unlabeled MGC and their newly generated daughter cells were easily recognized from the pre-labeled neurons. Cells were then fixed and analyzed by immunocytochemical methods and RQ-PCR.

In the diverse cellular culture protocols described above, the number of cells seeded in each culture dish was standardized; in each experiment we seeded about 700,000 cells per dish (700,979 cells/dish; +/−49,755), so the starting points were comparable.

### Immunocytochemical and Immunohistochemical Methods

To prepare retina cryosections, PND 9, PND 14 and PND30 (adult) *wt* and *rd1* mice were sacrificed, their eyes were enucleated, and then fixed for 3 h. in PBS-2% PF at 4°C. After lens removal, eyes were immersed in PBS-30% sucrose until sunk, mounted in OCT blocks, frozen first at −20°C and then stored at −70°C until they were cut with a Leica CM1860 cryostat. Cultures were fixed for 1 h. with PBS-4%PF, followed by permeation with 0.1% Triton X-100. The cultures and slices were then stained at room temperature for 10 min with DAPI, TOPRO-3, to visualize nuclei, or for 30 min with phalloidin, to label the actin cytoskeleton. Alternatively, immunohistological staining of cells was carried out using an anti-CRX rabbit antibody (1:800), anti-NESTIN mouse antibody (1:100), anti-GS rabbit antibody (1:100), anti-BrdU mouse antibody (1:00), and anti-opsin (Rho4D2) mouse antibody (1:100). Secondary antibodies, conjugated with Cy2 or Cy3 (1:200 dilution) were then used. Background labeling was removed by washing with PBS prior to microscopy.

### Cell Identification

Müller glial cells were identified by their flat and irregular morphology and by immunocytochemistry, with polyclonal antibodies against GS (Simón et al., 2012, 2015; Figures 1a–e,k–o and Supplementary Figures S1f–j). PHRs and amacrine cells were the two main neuronal types present in neuronal cultures; they represented more than 98% of the cells and were identified by their morphology using phase-contrast microscopy, and by immunocytochemistry with monoclonal antibodies against CRX and syntaxin (HPC-1), respectively. Controls for immunocytochemistry were done by omitting the primary or secondary antibodies. PHRs have a round cell body of about 5−10 μm, and a short and single axon, which usually ends in a conspicuous synaptic “spherule”; sometimes these cells display a connecting cilium at the opposite end, but they fail to develop their characteristic outer segments (Figures 1f–j,k–o and Supplementary Figures S1a–e, S2b,f); all of them express CRX, the PHR specific transcription factor, and were identified by using a CRX antibody (Cheryl M. Craft’s laboratory; University of Southern California, Los Angeles, CA, United States), (Garelli et al., 2006; Figures 1h,l). Amacrine cells are larger than PHRs (10–30 μm) and have multiple neurites; they all show anti-syntaxin immunoreactivity (Barnstable, 1980), which starts at early stages of development, and is retained even after undergoing degenerative changes that alter their morphology (Politi et al., 2001; Figure 1g).

### Evaluation of Cell Proliferation

To identify cells that were replicating their DNA, mixed neuron-glial cultures were incubated with 50 μM BrdU (final concentration in culture) for the final 24 h. before fixation, at days 6 and 11 in culture. After fixation, BrdU uptake was analyzed by immunocytochemistry using an anti- BrdU monoclonal antibody.

### Flow Cytometry

For quantitative evaluation of NESTIN expression, MGC from 11-day neuron–glial mixed cultures were analyzed by flow cytometry. Single cell suspensions, previously filtered using a 32-μm pore-size nylon mesh, were fixed and then permeated with solutions A and B from Dako IntraStain kit. Cells were then successively stained with primary (NESTIN 1/100) and secondary (Cy2 anti-mouse 1/200) antibodies. Flow cytometry analyses were done with a FACSCalibur flow cytometer (BD Biosciences). Quantification of NESTIN-positive MGC by FACS was performed using FACSComp. A minimum of 10,000 events was analyzed for each sample.

### Real-Time Quantitative Polymerase Chain Reaction (RQ-PCR)

*Wild type* and *rd1* mice were sacrificed at PND 0 (birth), PND 2, PND 4, PND 8, PND 10 (when all cell types have been generated and early signs of degeneration appear), PND 20 (progressive stages of PHR degeneration), PND 40 (complete loss of PHR), and PND 180. Retinas from three different mice were used for each stage. Retinas were dissected, and total RNA was extracted with Quick-Zol reagent (Kalium Technologies), according to the manufacturer’s instructions. The amount of RNA was measured by spectrophotometry. Reverse transcription of total RNA was performed using the High-Capacity cDNA Reverse Transcription Kit. Quantitative PCR was done by SYBR Green real-time PCR methods. The relative mRNA expression was calculated using the quantification cycle method (Cq-method) (Bustin et al., 2009), with *Eef2* (Eukaryotic elongation factor) for normalization. All experimental conditions were processed in triplicate. RQ-PCR primers were specifically designed to amplify the following cDNAs:

m-*Bax* F(5′TACTCCCGTCCTACCTCAGC 3′) R(5′CGCGG GTACTAAATGAACGC 3′),
m-*Nes* F(5′CCCTTAGTCTGGAAGTGGCTAC 3′) R(5′GTGC TGGTCCTCTGGTATCC 3′),
m-*Sox2* F(5′TGGATAACTGTTCAGCCACCAA 3′) R(5′GGC GCAGTATCTCATCTGCT 3′),
m-*Vim* F(5′ATGCTTCTCTGGCACGTCTT 3′) R(5′AGCCA CGCTTTCATACTGCT 3′),
m-*Eef2* F(5′GACTCTGAGAATCCGTCGCC 3′) R(5′CACAC AAGGGAGTCGGTCAG 3′).

The efficiency was about 90% for each pair of primers.

### Quantitative Image Analysis

After performing immunocytochemical labeling, cultures and tissue cryosections were analyzed by phase contrast and epifluorescence microscopy, using a Nikon Eclipse E600 microscope with a C-C Phase Contrast Turret Condenser and a Y-FL Epi-Fluorescence Attachment or with a laser scanning confocal microscope (Leica DMIRE2) with a 63X-water objective. Phase photomicrographs were processed using the XnView image processor to give a differential interference contrast effect. Fluorescence intensities of the 8- or 16-bit image were analyzed after manually outlining regions of interest (ROI) with the software Fiji-Image J (NIH, Bethesda, MD, United States) (Schindelin et al., 2012). The average fluorescence intensity of a given ROI was measured within the stained positive regions of the cell, and the average fluorescence intensity of an area of the same size positioned over a region outside the cell was subtracted. In cryosections, the measurements were carried out on randomly chosen cells, selected from phase-contrast images obtained from three PND 14 mice per each condition. We then calculated the *Corrected Total Cell Fluorescence* (CTCF) as *Integrated Density - (Area of selected cells x Mean fluorescence of background readings.* To evaluate morphological changes of radial processes of MGC during the time course of the degeneration, we measured and compared the thickness of NESTIN-immunolabeled radial processes in both *wt* and *rd1* mice at PND 14. Ten measurements were performed for each condition. Data were evaluated by Student’s *t*-test and differences were considered significant at *p* < 0.05.

### Statistical Analysis

The results represent the average of at least three experiments ( ± SEM), unless specifically indicated, and each experiment was performed in triplicate. For cytochemical studies, 10 fields per sample, randomly chosen, were analyzed in each case. Each value represents the average of at least three experiments, with three or four dishes for each condition ± SEM, unless otherwise indicated. Statistical significance was determined by Student’s two-tailed *t*-test or by ANOVA followed by Tukey’s test with *p* < 0.05 considered significant.

## Results

### The Course of *rd1* Neuronal Death Was Similar *in vivo* and *in vitro*

**FIGURE 2:**
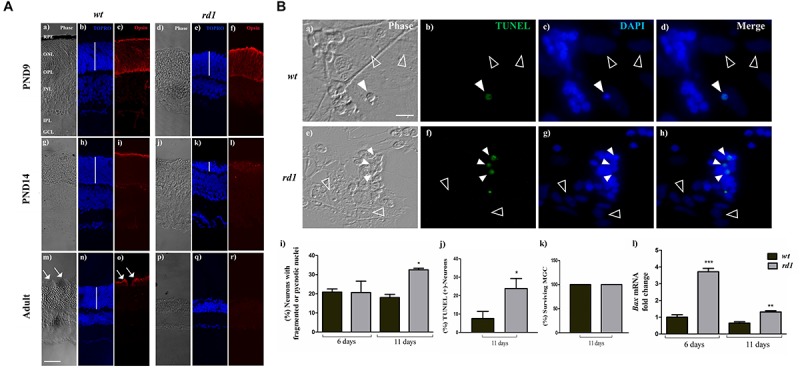
Selective degeneration of photoreceptors in the *rd1* mice retina and in mixed *rd1* neuron-glial cultures. Panel **(A)** phase **(a,d,g,j,m,p)** and fluorescence **(b,c,e,f,h,i,k,l,n,o,q,r)** photomicrographs of retinal cryosections from *wt* PND 9 **(a–c)**, PND 14 **(g–i)** and Adult (**m–o**, PND30); and *rd1* PND 9 **(d–f)**, PND 14 **(j–l),** and Adult **(p–r)**. Photoreceptors were labeled with an anti-opsin, Rho4-D2, antibody **(c,f,i,l,o,r)** and cell nuclei were stained with the nuclear probe TOPRO-3 **(b,e,h,k,n,q)**. White vertical bars show the thickness of the Outer Nuclear Layer (ONL) in *wt* and *rd1* retinas. Opsin immunolabeling was evident in *wt* and *rd1* retinas at PND 9 **(c,f)**, and concentrated in photoreceptor outer segments of *wt* retinas at PND 14 and 30 **(i,o)**; in contrast, it was barely visible in *rd1* retinas at PND 14 **(l)** and completely disappeared by PND 30 **(r)**. White arrows in **(m,o)** show outer segments in *wt* photoreceptors at PND 30. Panel **(B)** phase **(a,e)** and fluorescence **(b–d,f–h)** photomicrographs of mixed neuron-glial cultures from *wt* **(a–d)** and *rd1* **(e–h)** mice after 11 days in culture showing apoptotic neurons (white arrowheads) detected by TUNEL labeling **(b,f)**. Nuclei were stained with DAPI **(c,g).** Note that MGC nuclei (empty arrowheads in **a–h**) remained intact in *wt* and *rd1* cultures. Bars in **(i)** show the percentage of neurons with fragmented or pyknotic nuclei, **(j)** depict the percentage of TUNEL-positive neurons, **(k)** show the percentage of surviving MGC and **(l)** represent the fold-change in Bax mRNA levels relative to these levels in wt cultures at day 6. In Panel **(A)** scale bar: 50 μm. In Panel **(B)** scale bar: 5 μm. Results represent the mean ± SEM of three experiments (*n* = 3). Statistical analysis was performed using a two-tailed Student *t*-test [Panel **(B)** **j,k**] and a One-way ANOVA with a *post hoc* Tukey test [Panel **(B)** **i,l**]. ^*^p < 0.05, ^∗∗^*p* < 0.01, ^∗∗∗^*p* < 0.001.

We first compared the structure of *rd1* and *wt* retinas, at 9, 14, and 30 days of development *in vivo*. As expected, at PND 9 the outer nuclear layer (ONL) of both *wt* and *rd1* retinas had almost the same width and showed a similar pattern of opsin labeling (Figures 2Aa–f). In contrast, by PND 14 the ONL in *rd1* retinas was significantly reduced and showed a decrease in opsin labeling, which was below detectable levels after 4 weeks of development (Adult). In contrast, ONL width and opsin labeling were preserved in *wt* retinas (Figures 2Ag–r).

To investigate interactions between neurons and MGC, we prepared neuron-glial cultures (Figures 2Ba–h). Analysis of CRX labeling at 6 and 11 days *in vitro* revealed that most neurons in these mixed cultures were PHRs. In mixed *wt* cultures, PHRs amounted to 96 ± 29% and 94 ± 18.5%, respectively, of total neurons; similarly, in *rd1* cultures, these values were 94.5 ± 37% and 96 ± 20%, respectively, Supplementary Figure S2). The course of PHR death in mixed neuro-glial cultures was in close correspondence with the observed in whole retinas. By day 11 in culture (equivalent to 13 days *in vivo*), a marked increase in the amount of TUNEL-positive neurons (Figures 2Bb,f) and of pyknotic nuclei (Figures 2Bc,g, white arrowheads) was observed in *rd1* compared to *wt* neuronal cultures. Whereas at day 6 in culture (equivalent to 8 days *in vivo*) both *wt* and *rd1* cultures had similar percentages of pyknotic or fragmented neuronal nuclei, by day 11 the percentage of fragmented nuclei remained at 18 ± 3% in *wt* cultures but increased to 32.5 ± 1.7% in *rd1* cultures (Figure 2Bi). Consistently, the percentages of TUNEL-positive PHRs by day 11 were 7.5 ± 3.8% and 23.9 ± 5.4% in *wt* and *rd1*cultures, respectively (Figure 2Bj). In contrast, no TUNEL-positive MGC were observed at either time in culture; in addition, MGC had intact nuclei in the *rd1* and in the *wt* cultures (Figures 2Bb,c,f,g empty arrowheads). Quantitative analysis showed that the percentage of surviving *rd1* and *wt* MGC in each case was 100% ± 0.0%; (Figure 2Bk). We then evaluated the expression level of the Bcl-2 pro-apoptotic protein BAX in mixed neuron-glia cultures. The translocation of this protein to the outer mitochondrial membrane is crucial for triggering the intrinsic apoptotic pathway. Analysis of *Bax* mRNA levels by RQ-PCR showed they significantly increased (in arbitrary units) in *rd1* compared to *wt* cultures, with a 3.7 ± 0.4 fold-increase at day 6 and 1.3 ± 0.2 fold-increase at day 11 (Figure 2Bl).

### Cell Cycle Progression Decreased in *rd1* MGC

**FIGURE 3:**
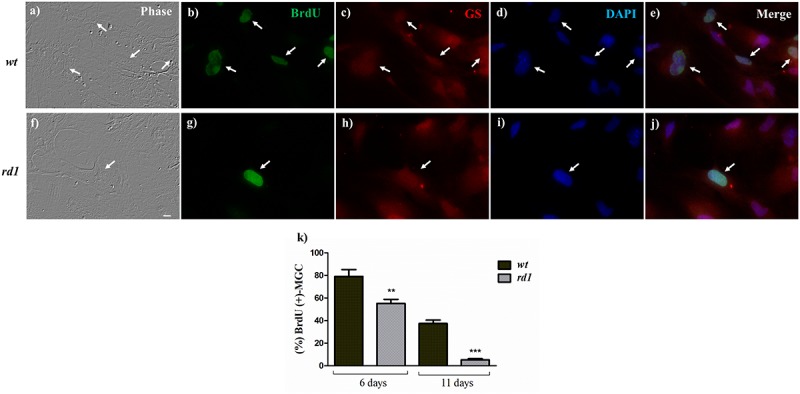
Decreased proliferation of *rd1* Müller glial cells in neuron-glial cultures. Phase **(a,f)** and fluorescence photomicrographs of 11-day *wt* **(a–e)** and *rd1* **(f–j)** mixed neuron-glial cultures showing BrdU uptake by MGC, determined with a monoclonal anti-BrdU antibody (**b,g**, arrows). MGC were identified by their labeling with an anti-GS antibody **(c,h)**. Nuclei were labeled with DAPI **(d,i)**. Merge **(e,j)**. Bars in **(k)** represent the percentage of BrdU-positive at days 6 and 11. Scale bar: 30 μm. Results represent the mean ± SEM of three experiments (*n* = 3). Statistical analysis was performed using a One-way ANOVA with a *post hoc* Tukey test. ^∗∗^*p* < 0.01, ^∗∗∗^*p* < 0.001.

Since one of the hallmarks of stem cells is their proliferative capacity, we evaluated whether it was affected in *rd1* MGC in mixed neuron-glial cultures (Figures 3a–j). Evaluation of BrdU uptake in mixed cultures showed that this uptake was lower in *rd1* than in *wt* MGC. By day 11, less BrdU-labeled MGC were visible in *rd1* than in *wt* cultures (Figures 3a,f). Quantitative analysis established that the percentages of BrdU-labeled MGC were significantly lower in *rd1* than in *wt* MGC (Figure 3k), both after 6 and 11 days in culture. By day 11, when *wt* cultures still had 37.36 ± 5.4% BrdU-positive cells, this percentage was only 5.2 ± 1.7% in *rd1* cultures (Figure 3k).

A reduction in proliferation in *rd1* MGC would lead to a progressive decrease in their number. Quantification of *rd1* and *wt* MGC evidenced that the number of *rd1* MGC was approximately half of that observed in *wt* cultures by day 6 (Table 1). This difference decreased with time in culture and tended to disappear by day 11; this might be a consequence of the cells reaching confluence in *wt* cultures, which posed additional restrictions to their proliferation. As a whole, the above results suggest that proliferation, a critical parameter of stem cells, was reduced in *rd1* MGC.

**TABLE 1:** Number of MGC per dish.

	**MGC/dish**	
**Days in culture**	***wt***	***rd1***
6	32375±7249	16720±1747^*^
11	104110±15110	95297±14693

Number of *rd1* and *wt* MGC per 35 mm dish after 6 and 11 *in vitro*. One-tailed Student *t*-test, ^*^p < 0.05.

### Stem Cell Markers Decreased in *rd1* MGC

**FIGURE 4:**
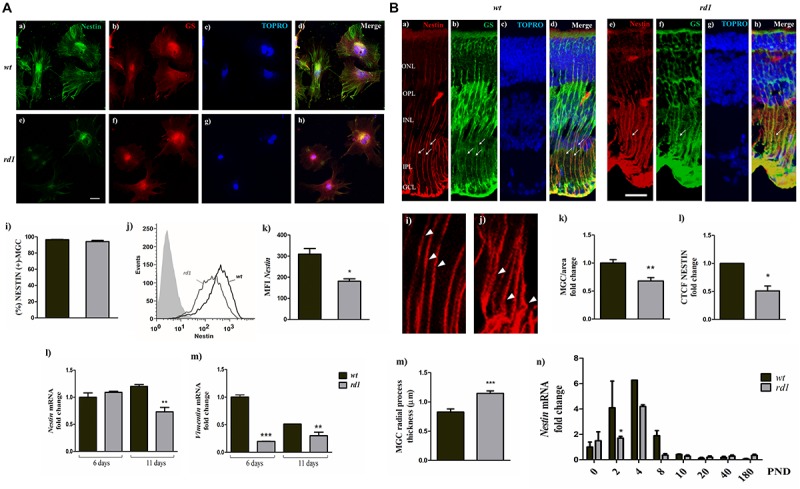
*Nestin* expression decreased in *rd1* retinas. Panel **(A)** fluorescence photomicrographs of 11-day *wt* **(a–d)** and *rd1* **(e–h)** mixed neuron-glial cultures showing NESTIN **(a,e)**; GS **(b,f)** and TOPRO-3 **(c,g)** labeling. Merge pictures are shown in **(d,h)**. Bars in **(i)** depict the percentage of NESTIN-expressing MGC, determined by flow cytometry. The histogram **(j)** shows the distribution of the intensity of NESTIN expression in MGC and bars in **(k)** show the medium fluorescence intensity of NESTIN labeling in *wt* and *rd1* MGC. Bars represent the fold-change in *Nestin* **(l)** and *Vimentin* mRNA levels **(m)** in *rd1* and *wt* cultures, relative to their levels in *wt* cultures at day 6. Scale bar: 40 μm. Results represent the mean ± SEM of three experiments (*n* = 3). Panel **(B)** Fluorescence photomicrographs of *wt* **(a–d)** and *rd1* **(e–h)** PND 14 retina cryosections showing MGC labeling with NESTIN **(a,e)** and GS **(b,f)**; MGC projections are indicated with thin arrows. Nuclei were stained with TOPRO-3 **(c,g)**. Merge pictures are shown in **(d,h)**. Confocal optical sections of retinas show the regular nestin filament distribution in MGC radial processes in *wt* retinas (**i**, arrowheads) and the conspicuous thickening and disorganization of radial processes at their basal ends in the *rd1* retinas (**j**, arrowheads). Bars in **(k)** show the fold change in the number of NESTIN-expressing MGC per area. Bars **(l)** depict the *Corrected Total Cell Fluorescence* (CTCF) as *Integrated Density*− *(Area of selected cells x Mean fluorescence of background readings*, analyzed after manually outlining regions of interest (ROI) with the software Fiji-Image J (see section “Materials and Methods”). Bars in **(m)** depict the thickness of MGC radial processes in *wt* and *rd1* retinas [shown with thin arrows in Panel **(B) a,b,d,e,f,h**; arrowheads in **i,j**]. Bars in **(n)** represent the fold changes in *Nestin* mRNA levels at PND 0-180 in *rd1* and *wt* mice retinas relative to their levels at PND 0 in *wt* retinas. Scale bar: 20 μm. Results in **k–m** represents the mean ± SEM of ten separate measures. Results in **(n)** represent the mean ± SEM of three mice per condition at each PND day analyzed. Statistical analysis was performed using a two-tailed Student *t*-test [Panels **(A) i,k** and **(B) k–m]** and a One-way ANOVA with a *post hoc* Tukey test [Panels **(A) l,m and (B) n]**. ^*^p < 0.05, ^∗∗^*p* < 0.01, ^∗∗∗^*p* < 0.001.

To investigate whether the stem cell potential of MGC in *rd1* retinas was affected, we evaluated the expression of *Nestin*, a neuroectodermal stem cell marker that is expressed in many different, mitotically active cell types during development (Lendahl et al., 1990; Park et al., 2010). By day 11, nearly all MGC in *wt* neuron-glial cultures had an intense NESTIN labeling, with a widespread, radial distribution toward the edges of the cells; by contrast, *rd1* MGC showed weaker NESTIN labeling, which remained close to the nucleus or formed fiber bundles (Figures 4Aa–h). Further analysis of NESTIN expression by flow cytometry showed that at day 11 the number of MGC expressing NESTIN was similar in both *wt* and *rd1* cultures (Figure 4Ai); however, evaluation of mean fluorescence intensity (MFI) evidenced that this intensity, i.e., NESTIN expression, was lower in *rd1* than in *wt* MGC (Figures 4Aj,k). This implies that although the amount of MGC expressing NESTIN remained similar in *rd1* and *wt* cultures, individual *rd1* MGC expressed less NESTIN than their *wt* counterparts.

We then evaluated the levels of *Nestin* mRNA in *rd1* and *wt* mixed neuron-glial cultures at days 6 and 11. At day 6, *Nestin* mRNA levels were similar in *rd1* and *wt* cultures; however, by day 11, they were significantly reduced in *rd1* compared with *wt* cultures (Figure 4Al).

Since NESTIN participates in the organization of intermediate filaments (Boraas et al., 2016), we also analyzed the expression of *Vimentin*, a type III intermediate filament protein. *Vimentin* mRNA levels showed a significant fold-reduction in *rd1* neuron-glial cultures compared with *wt* cultures, both by days 6 and 11 (Figure 4Am).

Similar changes in NESTIN expression were observed in MGC *in vivo.* Since the reduction in NESTIN expression was more evident at day 11 *in vitro*, we analyzed NESTIN distribution in cryosections from *wt* and *rd1* retinas at PND 14, an equivalent time of development *in vivo*. MGC showed an intense NESTIN labeling throughout the *wt* retina, which overlapped with GS labeling (Figures 4Ba–d). In contrast, NESTIN labeling was reduced and lost its fibrillary pattern in MGC in *rd1* retinas (Figures 4Be–h), and the number of NESTIN-labeled MGC per area decreased in *rd1* compared to *wt* retinas (Figure 4Bk). Quantitative analysis of fluorescence intensity evidenced a remarkable decrease in NESTIN immunolabeling in *rd1* retinas, which was reduced to half of that observed in *wt* retinas (Figure 4Bl). Conspicuous morphological changes were also evident in MGC in *rd1* retinas. The basal regions of MGC projections were thicker in *rd1* than in *wt* retinas, as observed when analyzing equivalent areas and positions in retinal sections (Figures 4Bi,j,m).

A maximum peak of mitosis occurs in newborn mice between PND 0 to PND 3 (Livesey and Cepko, 2001). As NESTIN has been proposed to play a role in the transition from proliferating stem cells to postmitotic neurons (Zimmerman et al., 1994), *Nestin* expression would be expected to increase at PND 3, the timepoint at which most proliferating stem cells in the retina become postmitotic and begin to differentiate as neurons (Livesey and Cepko, 2001). Analysis of the changes in *Nestin* mRNA levels in *wt* and *rd1* retinas at different developmental times evidenced that in *wt* retinas *Nestin* mRNA increased 4.1 ± 2.1 and 6.3 ± 0.01 fold at PND 2 and PND 4, respectively, compared to PND 0 (Figure 4Bn). By contrast, in *rd1* retinas *Nestin* mRNA at PND 2 remained at PND 0 levels, and by PND 4, its fold increase was lower than that observed in *wt* retinas (Figure 4Bn). By PND 8, the fold change in *Nestin* levels was below PND0 levels, both in *rd1* and *wt*, and remained lower than PND0 levels from PND 10 to adulthood.

**FIGURE 5:**
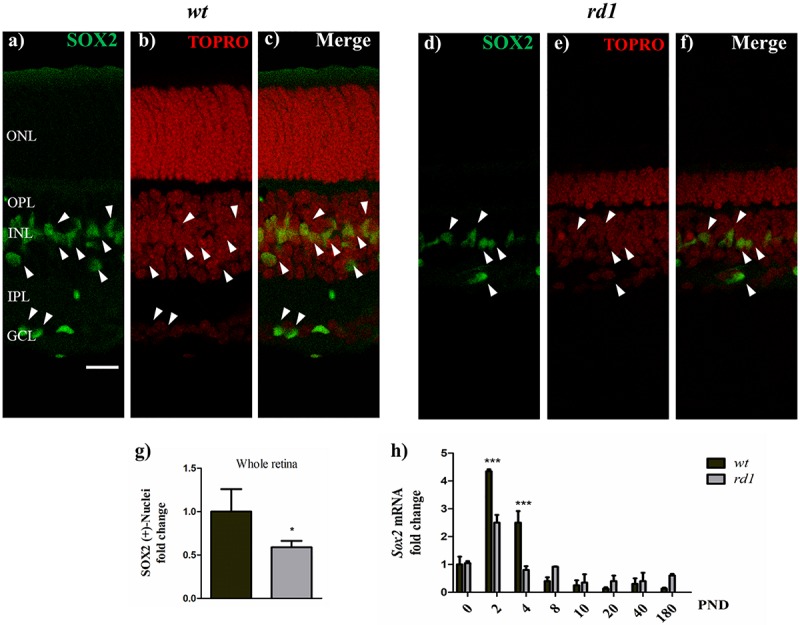
SOX2 expression decreased in *rd1* retinas. Fluorescence photomicrographs show SOX2 expression (**a,d**, arrowheads) in retina cryosections obtained from *wt* **(a–c)** and *rd1* **(d–f)** mice at PND14, determined with an anti-SOX2 polyclonal antibody. Nuclei were stained with DAPI **(b,e)** and merge pictures are shown in **(c,f)**. Bars **(g)** show the fold change of total SOX2-positive nuclei in *rd1* retinas relative to *wt* retinas. Bars **(h)** represent the fold changes in *Sox2* mRNA levels at PND 0-180 in *rd1* and *wt* mice retinas relative to their levels at PND 0 in *wt* retinas. Scale bar: 20 μm. Results in **(g)** represent the mean ± SEM of ten measures. Results in **(h)** represent the mean ± SEM of three mice per condition. Statistical analysis was performed using a two-tailed Student *t*-test **(g)**, and a One-way ANOVA with a *post hoc* Tukey test **(h)**. ^*^p < 0.05, ^∗∗∗^*p* < 0.001.

Expression of *Sox2*, a transcription factor involved in self-renewal of stem cells was also affected in *rd1* retinas. In *wt* retinas, *Sox2* mRNA levels peaked at PND 2, rapidly decreased at PND 4 and were below PND 0 levels beyond PND 8 (Figure 5h) whereas in *rd1* retinas, *Sox2* mRNA levels were significantly lower than in *wt* retinas at PND 2 and 4 (Figure 5h).

When PND 14 retina cryosections were analyzed, SOX2 expression was reduced in *rd1* compared to *wt* retinas (Figures 5a–f, arrowheads). At this time of development, *rd1* retinas had fewer SOX2-positive nuclei than *wt* retinas (Figure 5g). Whereas *wt r*etinas had SOX2-positive cells in the ganglion cell layer, virtually no SOX2-positive cells were detectable in this layer in *rd1* retinas.

### Interaction With *wt* Neurons Restored *Nestin* Expression in *rd1* MGC

**FIGURE 6:**
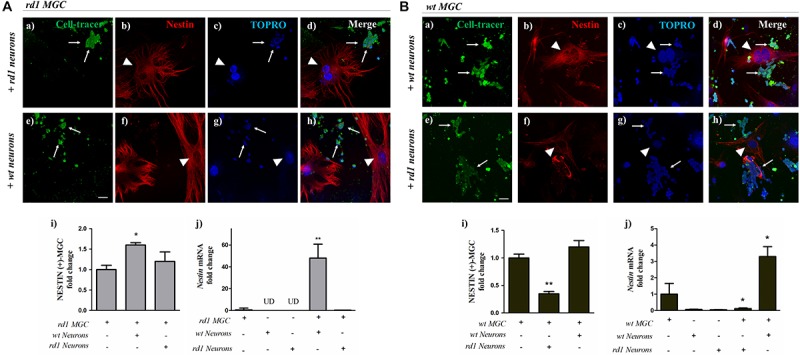
Changes in *Nestin* expression in MGC when co-cultured with *wt* or *rd1* neurons. Neuron-glial co-cultures were prepared seeding unlabeled *rd1* MGC (Panel **A**) or *wt* MGC (Panel **B**) over 3-day cultures of either *rd1* or *wt* neurons labeled with the green fluorescent CellTracer probe (thin arrows), and were then incubated for 24 h. Fluorescence photomicrographs show Cell-Trace labeled neurons (green in **a,e**) and NESTIN-labeled MGC (red in **b,f**, arrowheads); nuclei were stained with TOPRO-3 (blue in **c,g**). Merge pictures are shown in **(d,h)**. Bars in Panels **A (i)** and **B (i)** show the fold change in NESTIN-positive *rd1 MGC* (Panel **A**) *or wt MGC* (Panel **B**) when co-cultured with either *wt* neurons or *rd1* neurons, relative to pure *rd1* or *wt* MGC cultures, respectively. Bars in Panels **A (j)** and **B (j)** depict the fold change *in Nestin* mRNA levels in *rd1* MGC (Panel **A**) or *wt* MGC (Panel **B**) when co-cultured with either *wt* or *rd1* neurons, relative to pure *rd1 or wt* MGC cultures, respectively. Scale bar: 40 μm. Results represent the mean ± SEM of three experiments (*n* = 3). UD, undetected. Statistical analysis was performed using a One-way ANOVA with a *post hoc* Tukey test. ^*^p < 0.05, ^∗∗^*p* < 0.01.

As a whole, the above results show that proliferation and stem cell markers were decreased in *rd1* MGC, even before the PHR degeneration peak, suggesting a deficiency in the stem cell potential of these cells. We reasoned that the alterations in *rd1* neurons might impair neuron-glial crosstalk and affect the normal functions of *rd1* MGC, thus contributing to this deficiency. If this was the case, the interaction of healthy *wt* neurons with *rd1* MGC would, at least partially, reestablish their stem cell potential. Our culture systems allow us to prepare purified neuronal and MGC cultures, from developing *rd1* or *wt* retinas, and then seed them together to obtain co-cultures with different combinations of *rd1* and *wt* cells. To investigate the influence of neuronal cells on the stem cell capability of MGC, we separately cultured pure *rd1* and *wt* retinal neurons, labeled them with CellTracer^®^ for 3 days and then seeded unlabeled *wt* or *rd1* MGC over these neuronal cultures, to analyze NESTIN expression in the different neuron-glial co-cultures. *rd1* MGC co-cultured with *rd1* neurons had a faint NESTIN labeling (Figures 6Aa–d). Noteworthy, interaction with *wt* neurons (Figures 6Ae–h) increased NESTIN expression in *rd1* MGC. Quantitative analysis evidenced that NESTIN-positive *rd1* MGC significantly increased in *rd1* MGC-*wt* neuron co-cultures compared to *rd1* pure MGC cultures; in contrast, no significant change was observed in *rd1* MGC-*rd1* neuron co-cultures compared to *rd1* pure MGC cultures (Figure 6Ai). To further assess the effect of neuron-glial interactions on *Nestin* levels, we analyzed the levels of *Nestin* mRNA. In *rd1* MGC-*wt* neuron co-cultures, *Nestin* mRNA levels increased almost 50-fold relative to *rd1* pure MCG cultures; in contrast, no increase was observed in *rd1* MGC- *rd1* neuron co-cultures (Figure 6Aj).

If neuron-glial interactions were responsible for modulating the levels of stem cell markers such as *Nestin*, *rd1* neurons would downregulate these levels in *wt* MGC in co-culture. This reduction in NESTIN expression was evident when *wt* MGC were co-cultured with *rd1* neurons. Whereas *wt* MGC showed a bright NESTIN expression when co-cultured with *wt* neurons (Figures 6Ba–d), their NESTIN expression was noticeably reduced when co-cultured with *rd1* neurons (Figures 6Be–h). The fold change in NESTIN-positive *wt* MGC, relative to their number in *wt* pure MGC cultures, was reduced by half when co-cultured with *rd1* neurons; in contrast, NESTIN positive-*wt* MGC were the same in pure *wt* MGC cultures and in co-culture with *wt* neurons (Figure 6Bi). Noteworthy, the levels of *Nestin* mRNA were dramatically reduced in *wt* MGC-*rd1* neuron co-cultures relative to pure *wt* MGC cultures, and significantly increased, up to nearly 3.3 ± 1 times in *wt* MGC-*wt* neuron co-cultures (Figure 6Bj).

As a whole, these results suggest that the crosstalk between neurons and MGC is crucial for regulating Nestin expression in MGC, and interactions with *wt* neurons can restore this expression in *rd1* MGC.

### MGC Had a Distorted Morphology and Were Overloaded With Neurons in *rd1* Cultures

**FIGURE 7:**
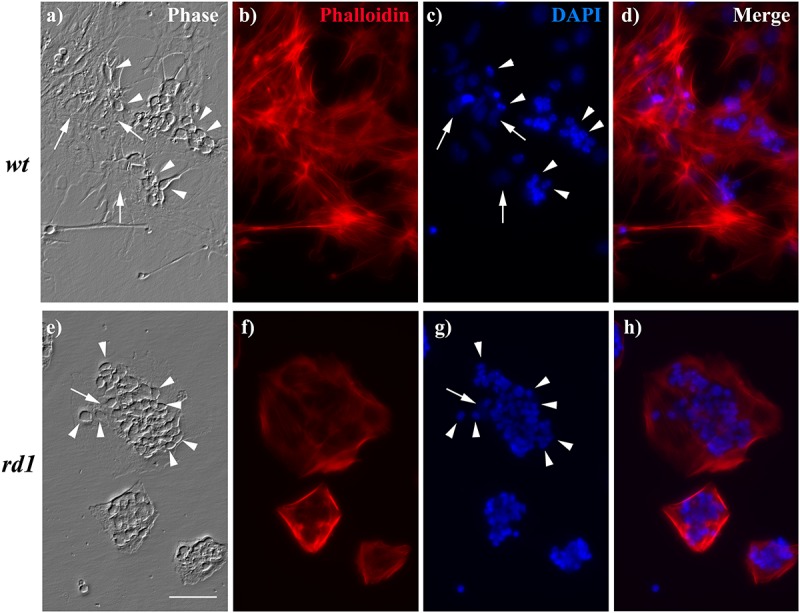
MGC morphology and neuron:glial ratio was altered in *rd1* mixed neuron-glial cultures. Phase **(a,e)** and fluorescence photomicrographs **(b–d,f–h)** of 6-day *wt* **(a–d)** and *rd1* **(e–h)** mixed neuron glial cultures showing neuronal cells (arrowheads) growing on top of MGC (arrows). Actin cytoskeleton was stained with phalloidin **(b,f)** and nuclei with DAPI **(c,g)**. Merge pictures are shown in **(d,h)**. Scale bar: 50 μm.

We finally analyzed whether the changes in the levels of intermediate filaments, as NESTIN and VIMENTIN, in *rd1* MGC correlated with changes in MGC morphology. Analysis of their actin cytoskeleton revealed that *rd1* MGC had remarkable alterations in their morphology. By day 6, *wt* cultures had many MGC, which exhibited an extended actin cytoskeleton (Figures 7a,b,d). In contrast, *rd1* cultures showed fewer MGC, which evidenced a marked retraction in their actin cytoskeleton and almost a complete loss of their lamellipodia, leading to a decrease in cell body size and a distorted morphology (Figures 7e,f,h).

In addition, the neuron:MGC ratio was markedly different in *wt* and *rd1* neuro-glial cultures. Whereas in *wt* cultures few neurons were observed growing on MGC (Figures 7a–d), their number markedly increased in *rd1* cultures by day 6 (Figures 7e–h). This difference might result from the reduction in the number of MGC in *rd1* cultures compared with *wt* cultures at this time of development, due to the decrease in their proliferation, as described above (Table 1). Quantitative analysis showed that the ratio of neurons per glial cell at day 6 was almost three-fold higher in *rd1* than in *wt* cultures (Table 2). This implies that individual *rd1* MGC supported significantly more neurons than their *wt* counterparts.

**TABLE 2:** MGC: neuron ratio.

	**G:N**	
**Days in culture**	***wt***	***rd1***
6	1:2.3±0.5	1:6.6±2.5^*^
11	1:1±0.3	1:2±0.5

MGC: neuron ratios in *w*t and *rd1* mixed neuron-glial cultures at 6 and 11 days *in vitro*. One-way ANOVA with a *post hoc* Tukey test, ^*^p < 0.05.

## Discussion

The major conclusions from the present work are that *rd1* MGC have a significant decrease in their stem cell markers even before the degeneration of PHR started in *rd1* retinas. Furthermore, at least one of their stem cell characteristics was restored by their interaction with *wt* neurons in co-culture, suggesting that a defective cross talk between neurons and MGC is at least partially responsible for this decrease.

The mechanisms regulating the neurogenic potential of MGC remain enigmatic. Their regenerative capacity is remarkably dissimilar among vertebrates. Although in the zebrafish, MGC can efficiently regenerate the retina after different lesions (Sherpa et al., 2008, 2014; Lenkowski and Raymond, 2014; Ranski et al., 2018), these cells are unable to function as retinal progenitors in mammals (Goldman, 2014). Intriguingly, cumulative evidence suggests that mammalian MGC have the molecular mechanisms to accomplish retina regeneration. Different injuries trigger their proliferation; activation of MGC following oxidative damage induces their dedifferentiation and re-entry in the cell cycle (Abrahan et al., 2009); neurotoxic damage promotes proliferation and the expression of progenitor markers in retina MGC (Ooto et al., 2004), which differentiate as specific neuronal types (Das et al., 2006). A human embryonic stem cell line (hESC) can differentiate into retinal amacrine neurons and ganglion cells, and integration of the hESC-derived retinal progenitors with a degenerated mouse retina increases the expression of specific PHR markers (Lamba et al., 2006). Human induced pluripotent stem cells (hiPSC) have been shown to differentiate into retinal progenitors and recapitulate retinal development to form three-dimensional retina cups containing major retinal cell types, including PHR with an advanced differentiation and photosensitivity (Zhong et al., 2014), implying that the generation of functional PHR from stem cells is feasible in mammals. Stem cells in mammalian retinas, such as MGC, can be assumed to replace neurons lost during the life span of healthy retinas. However, the substantial differences in the genetic setup of hiPSCs and mature MGC likely contribute to the incapacity of the latter to regenerate PHRs during retina degeneration. The inability of MGC to regenerate the retina has been suggested to result from either intrinsic features of mammalian MGC or extrinsic properties of their “niche,” which interfere with the reprograming process of these cells (Xia and Ahmad, 2016). Analysis of cultured MGC from *rd1* retinas revealed that their cell cycle was downregulated, compared to *wt* MGC. By day 6, prior to the onset of PHR degeneration, the number of *rd1* MGC was about half of that of *wt* MGC in mixed neuron-glial cultures and their BrdU-uptake was markedly reduced; this uptake was virtually negligible at day 11 *in vitro* in *rd1* MGC, whereas *wt* MGC still retained their proliferative capacity at this time in culture. This is in close agreement with previous findings showing that there are significantly less MGC in the *rd1* retinas than in the corresponding *wt* retinas (Chua et al., 2013). The significant reduction in the mitotic cycle in *rd1* MGC might further diminish the already limited regenerative capacity of healthy MGC.

Expression of stem cell markers was also reduced in *rd1* MGC. NESTIN is a well-established stem cell marker; its levels increase in most stem cells in the central nervous system (Lendahl et al., 1990) and it is downregulated upon differentiation (Sahlgren et al., 2006). The levels and distribution of NESTIN were affected in *rd1* MGC compared to *wt* MGC. *Nestin* was down regulated in *rd1* MGC, which had lower mRNA levels than *wt* MGC by day 11. Even when a similar number of MGC expressed NESTIN in *rd1* and *wt* neuron-glial cultures, the level of NESTIN expression was significantly lower in *rd1* cultures. This was consistent with the significant reduction in NESTIN-positive MGC in whole *rd1* retinas compared to *wt* retinas by PND 14. Early during development, the rodent retina is enriched in NESTIN-expressing progenitors, which later differentiate as neuronal progenitors and, further during development, as MGC. PND 2 is a critical point in development, corresponding with the highest mitotic peak of PHR progenitors and with the onset of MGC differentiation, which reaches its maximum levels between PND 5 and 8 (Cepko et al., 1996; Nelson et al., 2011). Interestingly, the largest difference in the level of *Nestin* mRNA between *rd1* and *wt* retinas was visible at PND2, suggesting a reduction in the number of progenitors in *rd1* retinas. SOX2 is a critical transcription factor for reprograming MGC toward a proliferative mode, for inducing their transformation into pluripotent stem cells and for the maintenance of neural stem cells (Favaro et al., 2009; Gorsuch et al., 2017). *Sox2* was also downregulated in *rd1* retinas; the levels of *Sox2* mRNA were lower in *rd1* than in *wt* retinas early during development. *Sox2* changes were similar to those described for *Nestin*. In *wt* retinas, the fold changes in *Sox2* mRNA levels relative to PND 0 showed a peak at PND 2, and rapidly decreased thereafter. In the *rd1* retinas the pattern of changes in *Sox2* mRNA was similar to the observed in *wt* retinas, but their increase at PND 2 and 4 was significantly lower than in *wt* retinas. Consistently, *rd1* retinas had remarkably less SOX2-expressing nuclei than *wt* retinas by PND 14. VIMENTIN is also considered a stem cell marker, although less specific than *Nestin*, since its expression has been found not only in developing tissues, but also in differentiated tissues (Traub et al., 1985; Lendahl et al., 1990). *Vimentin* is expressed in human stem cells when cultured on mouse PA6 stromal cells (Gong et al., 2008), and in cells derived from stem cell spheres when acquiring a Müller glial phenotype (Coles et al., 2004). VIMENTIN is closely linked to NESTIN, which is required for its polymerization (Park et al., 2010); in turn, NESTIN, along with the mitosis promoting factor (MPF), are important for the phosphorylation-mediated disassembly of VIMENTIN during mitosis (Chou et al., 2003). Noteworthy, *Vimentin* mRNA levels were significantly lower in *rd1* than in *wt* neuron-glial cultures by days 6 and 11 in culture. As a whole, our present findings that *wt* MGC expressed stem cell markers like *Nestin*, *Sox2* and *Vimentin*, and proliferated in culture are consistent with previous work from our laboratory evidencing that cultured rat MGC proliferate actively and express PAX6 and NESTIN (Insua et al., 2008; Simón et al., 2012). Our results also suggest that the regenerative capacity still preserved by MGC in *wt* retinas was further decreased in *rd1* retinas. *Nestin*, *Sox2* and *Vimentin* levels were reduced in *rd1* retinas, particularly in MGC, and these cells also evidenced a decreased proliferation rate. These findings support a decline in the stem cell potential of MGC in *rd1* retinas.

Little is known regarding the alterations that *rd1* MGC undergo during retinal degeneration (Roesch et al., 2012). Most studies on retinitis pigmentosa, including those using the *rd1* mice, focus mainly on the alterations affecting PHRs, such as genetic mutations, cell death, or abnormal development or functionality (Pittler and Baehr, 1991; Arikawa et al., 1992; Chen et al., 1996). Moreover, among the over 100 mutations leading to retinitis pigmentosa, most affect PHRs. Only a few reported mutations are known to affect MGC, such as a mutation in *Cralbp*, altering retinoid association/dissociation to its ligand binding pocket, and consequently the visual cycle (Golovleva et al., 2003), and no evidences of mutations affecting the regenerative capacity of MGC have been reported for this pathology. Our data strongly suggests that a defective neuron-glial crosstalk is responsible for the impaired stem cell capacity of *rd1* MGC. *Nestin* levels dramatically increased when *rd1* MGC were co-cultured with *wt* neurons. In contrast, when *wt* MGC were co-cultured with *rd1* neurons, *Nestin* levels were markedly reduced. These experiments strongly suggest that the crosstalk with neurons regulates *Nestin* expression in MGC and the interaction with healthy PHR progenitors is crucial for regulating MGC stem cell potential. To our knowledge, this is the first report highlighting the relevance of neuron-glial crosstalk in supporting the stem cell potential of MGC. This is consistent with the hypothesis that the “niche,” i.e., the environment surrounding MGC, might be modified in the retina during neurodegenerative diseases. MGC and neurons are known to maintain an active crosstalk, or “molecular dialogue” in the retina. The occurrence of gliosis in MGC in the beta-PDE model, *rd10* mice, before the beginning of retinal degeneration, suggests this crosstalk between PHR and MGC starts very early during development (Dong et al., 2017). Many of the factors involved in this “dialogue” have been identified; MGC release several molecules, such as FGF, GDNF, DHA, and LIF, that rescue PHRs in different models of retinal degeneration (Faktorovich et al., 1990, 1992; LaVail et al., 1992, 1998; Rotstein et al., 1996; Cayouette et al., 1998; Chong et al., 1999; Frasson et al., 1999; Harada et al., 2002; Miranda et al., 2009; German et al., 2013). MGC also release molecular cues that regulate their own regenerative responses, reducing proliferation (Fischer et al., 2004; Zhao et al., 2014). In turn, PHRs regulate MGC functions, even while undergoing degeneration. PHR degeneration modulates the expression of *Neurotrophin* receptor genes in retinal MGC (Harada et al., 2000). PHRs have also been reported to release stress signals, the damage-associated molecular patterns (DAMPs) during degenerative processes of the retina, that orchestrate a broad neuroprotective response in MGC, including release of Leukemia Inhibitory Factor (LIF), a critical PHR survival factor (Hooper et al., 2018). A further putative signal might be glutamate, which PHRs release as a neurotransmitter; activation of NMDA receptors has been shown to increase proliferation and differentiation of hippocampal neural progenitor cells (Joo et al., 2007) and might have a similar effect on retinal MGC. The progressive degeneration of PHRs in the *rd1* mice might lead to a shortage of glutamate, among other factors, which might affect MGC stem cell capability. Further research is required to establish how neuron-glial crosstalk is regulated and modified along development or after retinal injuries to regulate MGC regenerative capacity.

## Conclusion

Replacement therapies using stem cells are currently intensely investigated for retina neurodegenerations and establishing the most adequate source of these stem cells is crucial for these therapies. Our results imply that the limited capacity of mammalian MGC for restoring lost PHRs is further impaired in degenerated retinas. This capacity might be improved by their crosstalk with “healthy” PHR partners, suggesting that it is possible to manipulate these stem cells to recover their proliferative potential by creating an adequate “niche.” Identifying the molecular cues required for this manipulation is an exciting challenge that deserves further investigation.

## Ethics Statement

All procedures concerning animal use were carried out in strict accordance with the National Institutes of Health Guide for the Care and Use of Laboratory Animals and the protocols approved by the Institutional Committee for the Care of Laboratory Animals from the Universidad Nacional del Sur (Argentina) (Protocols CICUAE-UNS: 058/2015, 059/2015, and 060/2015).

## Author Contributions

All authors contributed intellectually to the manuscript. YV, HV-M, MD, OG, VA-P, SZ, and AG performed the experiments and analyzed the data. YV involved in the all experiments. YV, OG, HV-M, and AT performed the cultures. HV-M and AT involved in the immunocytochemistry imaging. MD involved in the immunohistochemistry and image processing. VA-P involved in the RQ-PCR assays. SZ and MD involved in the Flow Cytometry assays. AG involved in the preparation of retina cryosections. CC interpreted and analyzed the data and reviewed the manuscript. YV, OG, and LP elaborated strategies for the experiments. YV, OG, NR, and LP interpreted the data and elaborated the manuscript. LP and OG conducted the research and wrote the manuscript.

## Conflict of Interest Statement

The authors declare that the research was conducted in the absence of any commercial or financial relationships that could be construed as a potential conflict of interest.

## Supplementary Material

FIGURE S1 Specificity of photoreceptors and glial markers in culture. Phase **(a,f)** and fluorescence **(b−e,g−j)** photomicrographs of *wt* neuronal cultures **(a−e)**, showing photoreceptors labeled with an anti-CRX antibody **(c,h)**, and of *rd1* MGC cultures **(f**−**j)**, showing MGC labeled with an anti-GS antibody **(b,g)**. Nuclei were visualized with DAPI **(d,i)**. Merge images **(e,j)**. Scale bar: 20 μm.

FIGURE S2 CRX-positive neurons in mixed neuron-glial cultures. Phase **(a,e)** and fluorescence **(b**−**d,f**−**h)** photomicrographs of 6-day mixed *wt* **(a**−**d)** and *rd1* **(e**−**h)** neuron-glial cultures showing photoreceptors labeled with anti-CRX antibody **(b f)**. Cell nuclei were visualized with DAPI **(c,g)**. Merge images **(d,h)**. Bars in **(i)** represent the percentage of CRX-positive neurons (photoreceptors) at days 6 and 11 in *wt* and *rd1* mixed neuron-glial cultures. Scale bar: 20 μm. Statistical analysis was performed using a One-way ANOVA with a *post hoc* Tukey test. No significant differences were observed between conditions.
