# Supplementary figures and images for: A Defective Crosstalk Between Neurons and Müller Glial Cells in the rd1 Retina Impairs the Regenerative Potential of Glial Stem Cells
Source: Front Cell Neurosci. 2019 Jul 25;13:334. doi: 10.3389/fncel.2019.00334 (PMC6670004; doi:10.3389/fncel.2019.00334)

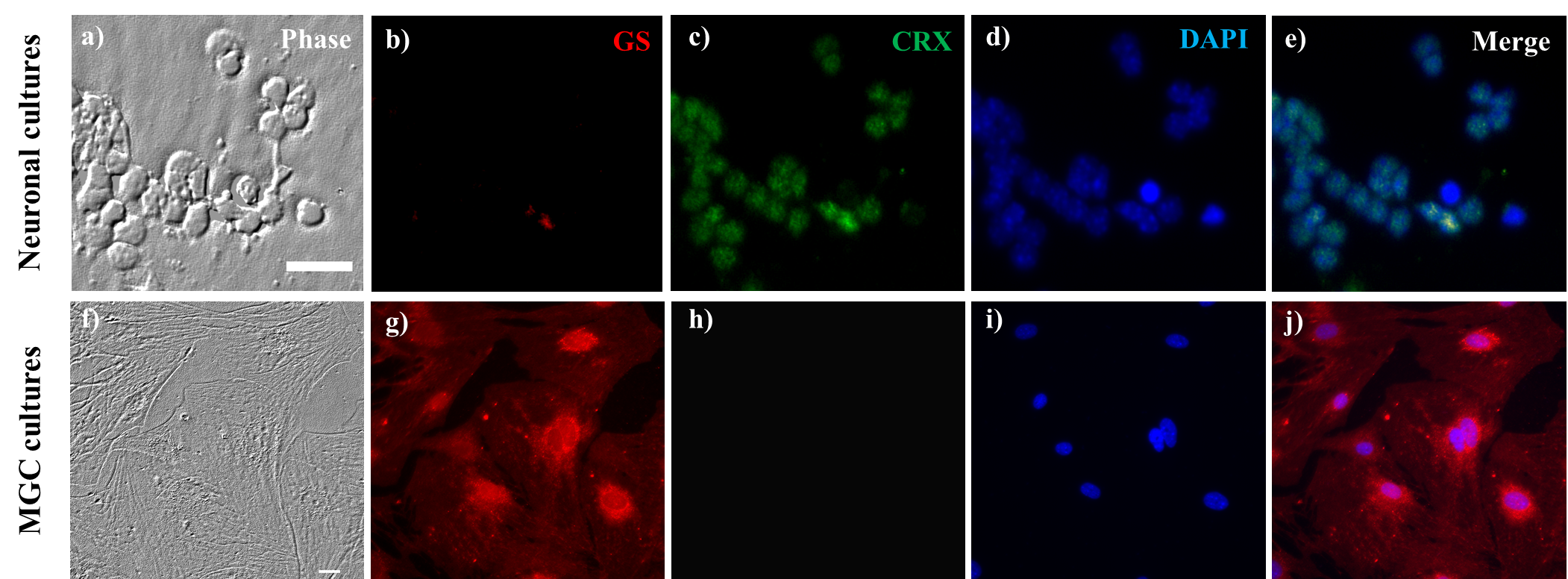

Supplement: FIGURE S1 — Specificity of photoreceptors and glial markers in culture. Phase (a,f) and fluorescence (b−e,g−j) photomicrographs of wt neuronal cultures (a−e), showing photoreceptors labeled with an anti-CRX antibody (c,h), and of rd1 MGC cultures (f−j), showing MGC labeled with an anti-GS antibody (b,g). Nuclei were visualized with DAPI (d,i). Merge images (e,j). Scale bar: 20 μm. [file Image_1.TIF]

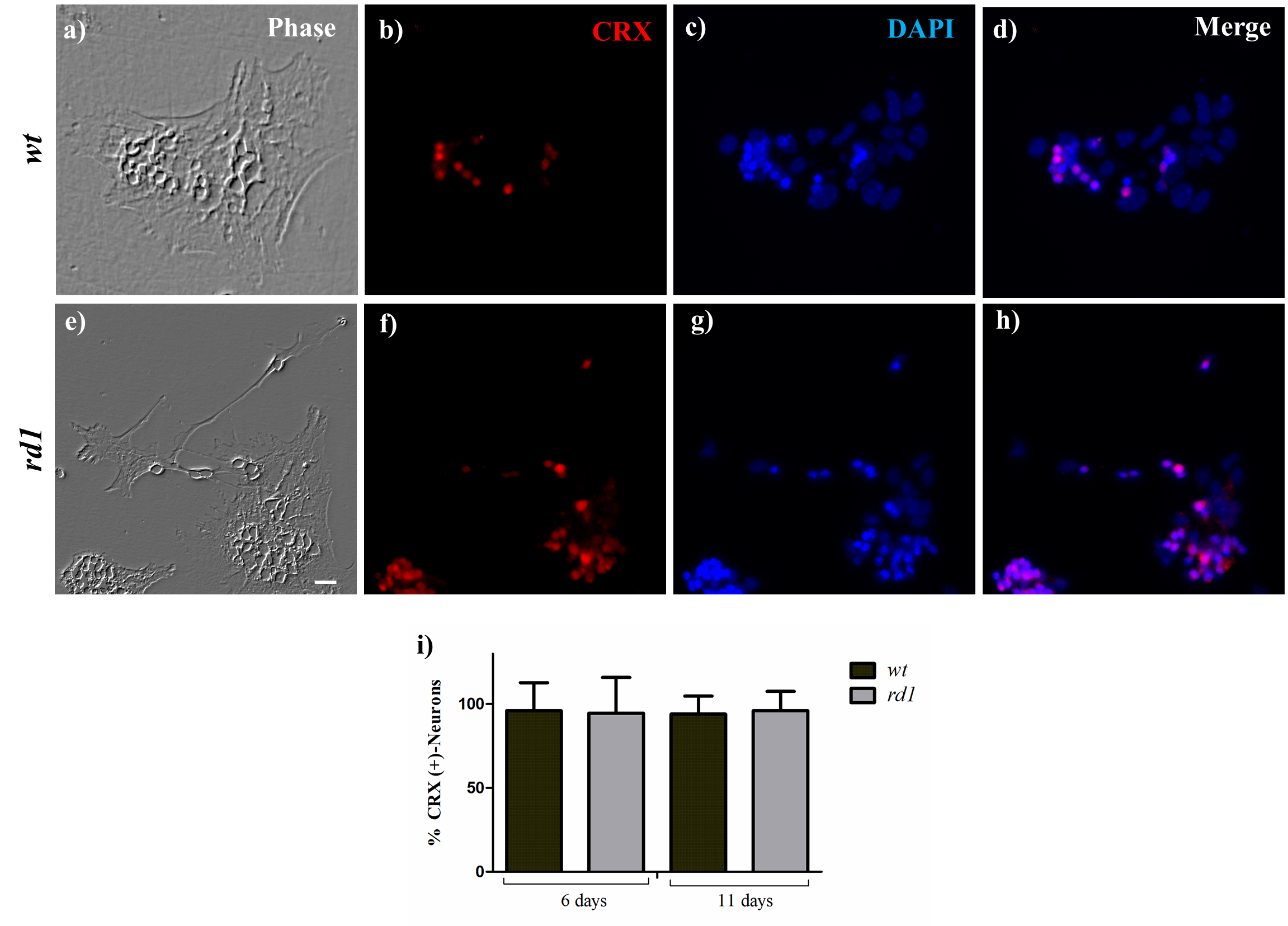

Supplement: FIGURE S2 — CRX-positive neurons in mixed neuron-glial cultures. Phase (a,e) and fluorescence (b−d,f−h) photomicrographs of 6-day mixed wt (a−d) and rd1 (e−h) neuron-glial cultures showing photoreceptors labeled with anti-CRX antibody (b f). Cell nuclei were visualized with DAPI (c,g). Merge images (d,h). Bars in (i) represent the percentage of CRX-positive neurons (photoreceptors) at days 6 and 11 in wt and rd1 mixed neuron-glial cultures. Scale bar: 20 μm. Statistical analysis was performed using a One-way ANOVA with a post hoc Tukey test. No significant differences were observed between conditions. [file Image_2.TIF]
